# Effect of upper limb, lower limb and combined training on health-related quality of life in COPD

**DOI:** 10.4103/0970-2113.59260

**Published:** 2010

**Authors:** Subin, Vaishali Rao, V. Prem, Sahoo

**Affiliations:** *West Fort Hi Tech Hospital, Pookunnam, Thrissur, India*; 1 *Department of Physiotherapy, Kasturba Medical College, Mangalore (A Constituent Unit of Manipal University), India*; 2 *Department of Pulmonary Tuberculosis and Chest Diseases (PTCD), Kasturba Medical College, Mangalore (A Constituent Unit of Manipal University), India*

**Keywords:** Chronic obstructive pulmonary disease, health-related quality of life, lower limb training, upper limb training

## Abstract

**Objectives:**

To study the effect of unsupported upper limb and lower limb exercise training and their combined influence on the exercise performance and health-related quality of life in COPD patients.

**Materials and Methods:**

Thirty patients were randomly assigned to one of the three groups, through block randomization. Of the three groups, group A received upper limb training, group B received lower limb training, and group C received both upper and lower limb training. Patients in group A, B, and C underwent exercise training five times a week for four weeks. The outcome measures used in the study were unsupported upper limb endurance test (UULEX), Six-Minute Walk Test (6-MWT), and a Chronic Respiratory Questionnaire. Statistical analysis was performed with analysis of variance, Wilcoxon scale, and a Kruskal Wallis one way ANOVA test, and a * P* value of .05 was used in the study.

**Conclusion:**

The combined upper limb and lower limb training group showed a significant improvement in the exercise performance and health-related quality of life.

## INTRODUCTION

Chronic Obstructive Pulmonary Disease (COPD) is a major cause of chronic morbidity and mortality throughout the world.[1] For patients with COPD, degradation in lung function is progressive, leading to premature disability and death. As the lung function decreases, the ability to engage in activities of daily living decreases, and thus the quality of life is impaired.[2]

In India it is one of the most common lung disorders following pulmonary tuberculosis. In most nontubercular chest clinics in India COPD constitutes nearly 25-30% of the cases.[3]

The upper extremities play an important role in many activities of daily living such as bathing, dressing, hanging out the wash, and gardening.[4] Patients with COPD frequently experience marked dyspnea and fatigue when performing these simple tasks.[5] Upper limb activities commonly require unsupported arm exercise, which poses a unique challenge for patients with COPD, whose upper limb muscles are required to act as accessory muscles of respiration. During unsupported arm exercise, the participation of the accessory muscles in ventilation decreases, and there is a shift of respiratory work to the diaphragm. This is associated with thoracoabdominal dyssynchrony, severe dyspnea, and termination of exercise at low workloads, especially in patients with more severe bronchial obstruction.[6]

The effectiveness of lower limb (LL) exercise training for patients with COPD has been well documented, with consistent and clinically significant improvements in exercise capacity, symptoms, and quality of life.[7]

It has been seen that upper limb exercise training for patients with COPD increases upper limb work capacity, improves endurance, and reduces oxygen consumption at a given workload.[8–10]

The benefits of combined upper limb and lower limb training, however, are less well defined. Therefore, there exists a need to measure the exercise performance and the functional outcome by combining unsupported upper limb exercises with lower limb exercises. The objective of this study is to find the effect of upper limb and lower limb exercise training on the functional outcome of patients with COPD.

## MATERIALS AND METHODS

The present study was conducted in the Department of Pulmonary Tuberculosis and Chest Disease, Kasturba Medical College, Mangalore. Thirty patients were randomly assigned to one of the three groups. Each patient underwent a formal evaluation program, including pulmonary function tests (PFTs), prior to the study. Pulmonary function testing was performed as per the standards outlined by the American Thoracic Society.[11] Patients received optimal medical therapy and were clinically stable at the time of their entry to the rehabilitation program.

The inclusion criteria were, patients should be in the age group of 45-75 years, FEV_1_ 45-75% of the predicted value, and they should not have been involved in any exercise program for the past one month. The patients were not included if they had had exacerbation within the past one month. Outcome measures used in the study were unsupported upper limb endurance test (UULEX),[12] Six-Minute Walk Test, and Chronic Respiratory Questionnaire.[13]

### Procedure

Thirty-four patients were referred for Pulmonary Rehabilitation. Four patients were excluded. Of them two had cervical spondylosis, one had vertigo, and the other had coronary heart disease. Thirty patients were randomly assigned to one of the three groups through block randomization. Of the three groups, group A received upper limb training, group B received lower limb training, and group C received both upper limb and lower limb training. Patients in group A, B and C underwent exercise training five times a week for four weeks.

All the patients were taught diaphragmatic breathing and pursed lip breathing exercises. After randomization of subjects to one of the three groups (A, B and C), the subjects underwent unsupported upper limb endurance test, six-minute walk test (6-MWT), and chronic respiratory questionnaire, before and after the training program.

**Figure 1:**
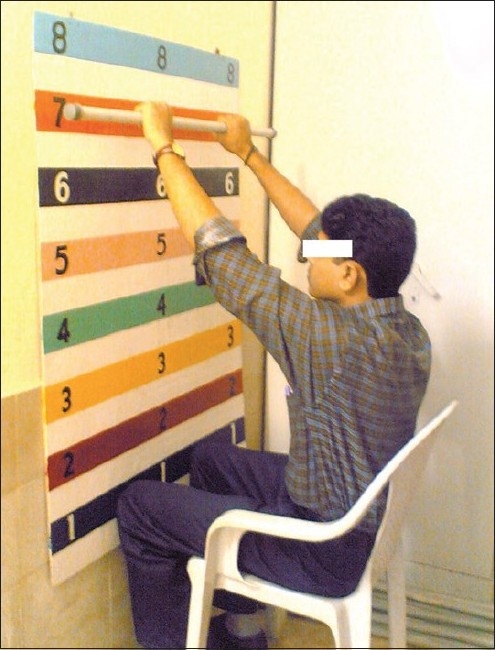
Maximum position reached during UULEX test

In our study the UULEX test was terminated if the patient experienced dyspnea or arm fatigue at the maximum position reached as shown in Figure 1. Duration of endurance level in the chart reached and the weight of the bar used were recorded. On the same day, the patient underwent a six-minute walk test, based on the guidelines of the American Thoracic Society.[14]

At the end of the UULEX and 6-MWT, dyspnea was recorded using a modified Borg 0-10 scale.[15] The patient filled the chronic respiratory questionnaire on the same day.

Patients in group A had 10 minutes of general warm up, progressive upper limb training, and 10 minutes of cool down.

Upper limb exercise training included,[10]

1. Throwing a ball against the wall with arms above horizontal in sitting position.
2. Passing a beanbag over the head in sitting position
3. Exercises on overhead pulleys in sitting position
4. Moving a ring across a wire without touching the wire, while arm was above horizontal.

Duration: Each exercise was performed for 40 seconds followed by 20 seconds rest. Exercises would be repeated four times in four minutes.

Group B had undergone 10 minutes of warm up, 20 minutes walking training, and 10 minutes of cool down.

Group C had undergone upper limb exercise training and lower limb exercise training on alternate days with the same protocol.

### Statistical analysis 

Statistical analysis was performed using the SPSS software for Windows (version 14.0). Alpha value was set as 05. Analysis of variance was used to test the differences among the demographic variables and PFT values. The Wilcoxon scale was used before and after training within groups. The Kruskal Wallis One Way ANOVA test was used to compare the measures in the ordinal scale and ratio scales between groups.

## RESULTS

Baseline characteristics of 30 male patients for three groups are shown in Tables 1 and 2. Table 3 represents pre and post difference following upper limb, lower limb and combined training on UULEX, six minute walk test and chronic respiratory questionnaire.

**Table 1:** Baseline data for demographic variables

Group	A	B	C	*P* value
Number	10	8	9	-
Age, y	59 ± 8	57 ± 8	60 ± 9	0.872
BMI, Kg/m^2^	19.6 ± 2	19 ± 3	20 ± 2	0.59
FEV_1_% predicted	40 ± 6	49 ± 11	46 ± 11	0.20
FEV_1_/FVC	66 ± 9	57 ± 10	63 ± 10	0.21
PEF	34 ± 9	38 ± 11	44 ± 8	0.12
Pack year	30 ± 10	35 ± 11	34 ± 11	0.58

*P value < 0.05, Comparison of groups at baseline showed no significant differences on forced expiratory volume in one second FEV_1_, body massindex BMI

**Table 2:** Baseline data for outcome variables

Group	A	B	C	*P* value
6MWD, meters	441 ± 63	463 ± 68	462 ± 78	0.65
UULEX, sec	235 ± 55	273 ± 104	253 ± 123	0.56
CRQ - DOMAINS				
Dyspnea (D)	2.87 ± 1.0	3.1 ± 0.7	3.17 ± 0.75	0.77
Fatigue (F)	3.23 ± 0.76	3.9 ± 0.79	3.51 ± 0.73	0.22
Emotion (E)	3.30 ± 0.58	3.7 ± 0.99	3.63 ± 1.48	0.70
Mastery (M)	29 ± 1.14	3.0 ± 0.93	3.50 ± 0.75	0.51

*P value < 0.05, Comparison of groups at baseline showed no significant differences for, six-minute walking distance (6-MWD), unsupported upper limb endurance time or CRQ

**Table 3:** Pre and post difference on UULEX, six-minute walk test and chronic respiratory questionnaire in group A, B and C

	Group A			Group B			Group C		
			
	Pre mean (±S.D)	Post mean (±S.D)	*P* value	Pre mean (±S.D)	Post mean (±S.D)	*P* value	Pre mean (±S.D)	Post mean (±S.D)	*P* value
UULEX	235.10	244.60	0.028*	273.25	272.75	0.888	253.33	263.22	0.042*
	(55.89)	(47.97)		(104.28)	(98.90)		(123.53)	(111.44)	
6 min	441.30	439.0	0.878	456.12	463.50	0.042*	462.11	479.44	0.03*
	(63.69)	(51.87)		(87.76)	(68.94)		(78.47)	(59.69)	
CRQ-D	2.87	3.32	0.005*	3.12	3.60	0.027*	3.17	3.82	0.006*
	(1.03)	(0.97)		(0.71)	(0.73)		(0.75)	(0.80)	
CRQ-F	3.23	3.91	0.005*	3.98	4.43	0.027*	3.51	4.22	0.006*
	(0.76)	(0.85)		(0.79)	(0.60)		(0.73)	(0.98)	
CRQ-E	3.30	3.78	0.007*	3.73	3.95	0.02*	3.63	3.94	0.010*
	(0.58)	(0.74)		(0.99)	(1.12)		(1.46)	(1.12)	
CRQ-M	2.99	3.93	0.008*	3.00	3.68	0.01*	3.50	4.16	0.008*
	(1.14)	(1.38)		(0.93)	(0.95)		(0.75)	(0.78)	

* *P* value < 0.05 is significant, UULEX - unsupported upper limb endurance test, 6min - six-minute walk test, and CRQ-D - Chronic Respiratory Questionnaire Dyspnea, CRQ-F - Chronic Respiratory Questionnaire Fatigue, CRQ-E - Chronic Respiratory Questionnaire Emotion, CRQ-M - Chronic Respiratory Questionnaire Mastery. Within group comparison of Group A and Group C, showed significant improvement on UULEX after training. Group B and C showed a significant improvement in six-minute walk test distance after training. Groups A, B, and C showed significant improvement in all the domains of CRQ, following training

## DISCUSSION

The baseline data of the demographic and outcome variables did not show any significant difference between the patient populations in the three groups, indicating the homogeneity of the population. Two patients from group B and one patient from group C did not attend for final assessment and could not be contacted.

In group A, the upper limb endurance was increased and six-minute walking distance was decreased. The present study showed an improvement in upper limb endurance time, which is in accordance with study done by Epstein *et al*., who found that arm training resulted in increased upper limb endurance.[16] This might be due to improved synchronization and coordination of accessory muscle action during unsupported arm activity.[4]

In group A, the six-minute walk test distance was decreased with a mean of 2 meters. Lake *et al*., compared arm training with leg training and found that training of the upper extremities improved arm function, but lower extremity capacity was decreased in that group. The training effect was specific for the muscle group trained, with no cross-over benefit seen between the arms and legs.[10]

The unsupported upper limb endurance test was terminated because of arm fatigue rather than breathlessness. Velleso *et al*., observed that activities such as screwing in a light bulb provoked greater dyspnea than the repetitive, stereotypic task of lifting pots.[17] He postulated that such coordination tasks are more dyspnea-inducing because of breath holding or a reduction in blood flow to the muscle during static contractions. The upper limb test did not incorporate a coordination component or static muscle contraction, which may explain the absence of dyspnea at the termination of the test.

In group B, the six-minute walk test was increased to a mean of 23 meters, whereas, the upper limb endurance time was decreased. Knox *et al*., demonstrated a significant improved result on the repeated performance of a 12-minute walking test over a four-week period.[18] The improvement in the six- minute walk distance was mainly due to aerobic training effects and specificity of training.[9] However, the distance covered in the present study was lesser than the suggested 54 meter lower limit of the clinical difference reported by Redelmeier *et al*.[19] The decrease in distance covered in the six-minute walk test might be due to the reduced duration of the training program.

In group C, the combined upper limb and lower limb training resulted in the improvement of both the upper limb and lower limb exercise capacity, which might be due to the specific training effects, which is in accordance with the previous studies.[4,8,10]

All the domains of CRQ were increased significantly pre- and post-training. Increase in the dyspnea domain might be due to the psychological benefits of exercise, which included increased motivation, desensitization to dyspnea,[7]and loss of fear of exercise. Reduction in anxiety led to an improved breathing pattern that could reduce dynamic hyperinflation, which in turn resulted in the improvement of the emotional domain. An increase in upper limb and lower limb endurance could be attributed to the improvement in the fatigue domain. The present study included breathing control training in all the groups, which resulted in an increase in the mastery domain.[19]

## CONCLUSION

Combined upper limb and lower limb training resulted in a significant improvement in the exercise performance and health-related quality of life.
